# Polycyclic aromatic hydrocarbons (PAHs) present in ambient urban dust drive proinflammatory T cell and dendritic cell responses via the aryl hydrocarbon receptor (AHR) *in vitro*

**DOI:** 10.1371/journal.pone.0209690

**Published:** 2018-12-21

**Authors:** Chelsea A. O’Driscoll, Madeline E. Gallo, Erica J. Hoffmann, John H. Fechner, James J. Schauer, Christopher A. Bradfield, Joshua D. Mezrich

**Affiliations:** 1 Department of Surgery, Division of Transplantation, School of Medicine and Public Health, University of Wisconsin-Madison, Madison, WI, United States of America; 2 Molecular and Environmental Toxicology Center, School of Medicine and Public Health, University of Wisconsin-Madison, Madison, WI, United States of America; 3 Wisconsin State Lab of Hygiene, Madison, WI, United States of America; 4 Civil and Environmental Engineering, College of Engineering, University of Wisconsin-Madison, Madison, WI, United States of America; 5 McArdle Laboratory for Cancer Research, School of Medicine and Public Health, University of Wisconsin-Madison, Madison, WI, United States of America; University of Louisville School of Medicine, UNITED STATES

## Abstract

Atmospheric particulate matter (PM) is a complex component of air pollution that is a composed of inorganic and organic constituents. The chemically-extracted organic fraction (OF) of PM excludes inorganics but retains most organic constituents like polycyclic aromatic hydrocarbons (PAHs). PAHs are ubiquitous environmental toxicants and known aryl hydrocarbon receptor (AHR) ligands. The AHR is a ligand activated transcription factor that responds to endogenous ligands and exogenous ligands including PAHs. Activation of the AHR leads to upregulation of cytochrome P450 (CYP) metabolizing enzymes which are important for the biotransformation of toxicants to less toxic, or in the case of PAHs, more toxic intermediates. Additionally, the AHR plays an important role in balancing regulatory and effector T cell responses. This study aimed to determine whether PAHs present in PM aggravate inflammation by driving inflammatory T cell and dendritic cell (DC) responses and their mechanism of action. This study tests the hypothesis that PAHs present in PM activate the AHR and alter the immune balance shifting from regulation to inflammation. To test this, the effects of SRM1649b OF on T cell differentiation and DC function were measured *in vitro*. SRM1649b OF enhanced Th17 differentiation in an AHR and CYP-dependent manner and increased the percent of IFNγ positive DCs in an AHR-dependent manner. SRM1649b PAH mixtures enhanced Th17 differentiation in an AHR-dependent but CYP-independent manner and increased the percent of IFNγ positive DCs. Cumulatively, these results suggest that PAHs present in PM are active components that contribute to immune responses in both T cells and BMDCs through the AHR and CYP metabolism. Understanding the role of AHR and CYP metabolism of PAHs in immune cells after PM exposure will shed light on new targets that will shift the immune balance from inflammation to regulation.

## Introduction

In recent years, air pollution has emerged as the leading cause of death and disease worldwide and when considering all human pathology related to the environment, air pollution is the primary offender [1]. According to the World Health Organization (WHO) Air Quality Guideline, 95% of the world’s populations live in areas that exceed the recommended guideline for ambient air pollution [1]. Atmospheric particulate matter (PM) is a component of air pollution, and is the largest environmental risk factor for premature death worldwide [1]. PM is a complex heterogenous mixture that contains several chemical components each generated from multiple sources [2]. Additionally, the composition of PM varies over time and by location [2]. Despite this complexity and variability, all atmospheric PM is currently regulated by total mass based on the assumption that all PM is equally toxic [3, 4]. This regulatory strategy assumes all PM of the same mass will elicit the same biological effects and ignores individual characteristics and components [3, 4].

A long-standing gap in knowledge remains as to whether all PM of the same mass elicit the same biological effects or if individual components and characteristics of PM drive the aggravated responses. The adverse human health effects of PM have been well established, but the specific characteristics or components of PM that are harmful are not well understood. Research efforts have been made to identify the active component(s) of PM responsible for worsening disease by testing the effects of the intact PM, the chemically-extracted organic fraction (OF) adsorbed to it, or individual components of PM. The chemically-extracted OF excludes metals and inorganics but retains most organic compounds, including toxicants such as polycyclic aromatic hydrocarbons (PAHs), 16 of which are classified by the United States (U.S.) Environmental Protection Agency (EPA) as priority pollutants [5, 6]. PAHs are ubiquitous environmental toxicants and known aryl hydrocarbon receptor (AHR) ligands [7]. Based on this, we hypothesize PAHs present in PM may contribute to worsened disease states.

The AHR is a ligand-activated transcription factor that responds to endogenous ligands in addition to exogenous xenobiotic ligands, such as PAHs [8, 9]. Upon ligand binding, the AHR activates xenobiotic metabolizing enzymes: cytochrome P450 (CYP) 1A1, 1A2, and 1B1 [8–10]. CYP enzymes are phase 1 metabolizing enzymes responsible for catalyzing oxidative biotransformation of xenobiotics [11]. This biotransformation can make compounds less toxic but has the potential to generate reactive intermediates as is the case with PAHs [12, 13]. Some PAHs have been shown to inhibit CYP enzymes altering their own metabolism as well as the metabolism of other PAHs [14, 15].

The AHR plays a critical role in modulating effector and regulatory T cell responses [16, 17]. More specifically, some AHR ligands have been shown to drive forkhead box 3 (FOXP3)^+^ regulatory T cell (Treg) differentiation and others have been shown to enhance proinflammatory T helper (Th)17 differentiation *in vitro* and *in vivo* [16, 17]. Recently, the extent and duration of AHR activation has been shown to be critical in shifting the balance from regulatory responses to proinflammatory responses [18]. Increased Th17 differentiation could play a role in inflammatory disease states including PM-induced autoimmunity. Previously our lab demonstrated that the intact PM of an ambient urban dust sample, SRM1649b, increased IL-17 and CYP1a1 mRNA levels in the lung of mice exposed via intranasal instillation for 3 days [19]. In addition, an increase in IL-17 and gamma interferon (IFNγ) protein levels were measured in a mixed leukocyte culture which interrogates the effects of SRM1649b PM on dendritic cells (DCs) and T cells in an alloimmune setting where cells from C57BL/6J mice are stimulated with DCs from Balb/c mice [19]. Moreover, the chemically-extracted OF elicited the same level of enhanced Th17 differentiation and AHR activation as the intact PM suggesting the active component driving Th17 differentiation was present in the OF [19]. In addition, diesel exhaust OF and cigarette smoke OF, both of which contain PAHs, enhanced Th17 differentiation *in vitro* [19]. More recently, we demonstrated that synthetic PAH mixtures based on two diesel exhaust PM samples enhanced Th17 differentiation dependent on the AHR and/or CYP metabolism [20].

The goal of the current study was to determine whether individual components of PM, specifically PAHs, aggravate inflammation by interrogating the effects of PM derivatives on T cells and antigen presenting cells (APCs). Additionally, we sought to identify specific pathways through which PAHs present in PM enhance proinflammatory responses. We hypothesized that PAHs present in PM activate the AHR and alter the immune balance shifting from regulation to inflammation. More specifically, PAHs alter the immune balance by enhancing Th17 differentiation and generating proinflammatory bone marrow dendritic cells (BMDCs) and ultimately aggravate inflammatory states. To test this, the effects of SRM1649b OF on T cell differentiation and APC function were measured *in vitro*. SRM1649b OF enhanced Th17 differentiation in an AHR and CYP-dependent manner, suggesting upregulation of CYPs and metabolism of PAHs are important in driving Th17 responses. Additionally, SRM1649b OF increased the percent of IFNγ positive BMDCs in an AHR-dependent manner. To address whether PAHs present in PM were driving proinflammatory T cell and BMDC function, a PAH mixture that replicates the milieu of PAHs present in SRM1649b OF was synthesized. The synthetic SRM1649b PAH mixture enhanced Th17 differentiation in an AHR-dependent manner, but a CYP-independent manner, suggesting that for the PAHs alone, CYP metabolism is not driving Th17 differentiation. Similar to SRM1649b OF, the synthetic SRM1649b PAH mixture increased the percent of IFNγ positive BMDCs. Cumulatively, these results suggest that PAHs present in PM are active components that contribute to immune responses in both T cells and BMDCs via the AHR. These results illustrate the complicated nature of PM samples and the need for understanding how individual components and mixtures of those components alter the immune response and identification of specific pathways that lead to regulatory responses versus effector responses.

## Materials and methods

### Mice

Wild-type (WT), C57BL/6J mice were obtained from Jackson Laboratories (stock# 000664) or bred in house in a Specific Pathogen Free facility. Christopher Bradfield provided *Ahr* null (*Ahr*^-/-^) [21] and *Cyp1a1*^-/-^*Cyp1b1*^-/-^*Cyp1a2*^-/-^ (*CypTKO*) mice on a C57BL/6J background. All these genotypes have been backcrossed into the C57BL/6J background for eight generations, ensuring that the knockout genotypes reside in a genetic background that is >99.8% C57BL/6J [22]. All mice were maintained under specific, pathogen free conditions. All animal experiments were performed in accordance with protocols approved by the School of Medicine and Public Health Institutional Animal Care and Use Committee at the University of Wisconsin-Madison (Protocol Number: M005939). All mice were anesthetized using isoflurane anesthesia, and all efforts were made to minimize suffering.

### Organic fraction (OF) extraction

The organic fraction of SRM1649b PM sample was isolated by extracting the National Institute of Standard and Technology (NIST) SRM1649b PM sample with methylene chloride (DCM) using a Soxhlet extractor. PM samples that were collected as powders (i.e. SRMs) were placed in cellulose thimbles to contain the sample. The methylene chloride extraction was conducted for 24 hours with nominally 5 solvent cycles per hour. The extracts were then reduced in volume by evaporating the solvent by directing a high-purity, gaseous nitrogen stream into the sample, which was held at 50°C in a water bath. The solvent evaporation continued until the extract volume reached 0.1mL. 1.0mL of hexane was then added to the concentrated extract and the volume was reduced by the nitrogen blow down to 0.1mL. This process was repeated twice to transfer the extract into hexane and remove the methylene chloride. After the three hexane transfers, 0.2 ml of dimethyl sulfoxide (DMSO) was added the residual hexane was evaporated with nitrogen under the same conditions for 30 minutes. Additional DMSO was then added to obtain the final extract concentration.

### Preparation of synthetic PAH Mixtures replicating the PAH milieu of SRM1649b

Representative PAH mixtures of SRM1649b were made by creating a synthetic mixture of 15 standard PAHs at concentrations similar to SRM1649b. The PAH mixture is a synthetic mixture that replicates the milieu of 15 PAHs present in SRM1649b. SRM1649b is composed of many other PAHs aside from the 15 chosen from this study (S1 Table), but these PAHs were chosen as most are on the U.S. EPA list of priority pollutants [23]. Additionally, of the PAHs characterized in SRM1649b, the 15 PAHs chosen are present at high concentrations and rank in the top 40, most within the top 20, based on mass fraction. Individual PAH standards were purchased from AccuStandard (Z-013-SET). All samples were prepared by the WSLH (Madison, WI). The standards were transferred into a centrifuge tube and the solvent (DCM or MeOH) was blown down under nitrogen in a 50°C water bath until 0.1mL remained. Approximately 2mL of hexane was added and blown down under nitrogen in a 50°C water bath until 0.1mL remained. This process was repeated three total times to ensure all the DCM was gone and the final solution was in hexane only. The appropriate amount of DMSO was added and the tube was placed in the 50°C water bath under nitrogen until all hexane was removed and the PAHs were left in DMSO.

### Isolation of naïve T cells and T-cell differentiation

Naïve CD4^+^ T cells were isolated by negative selection and purified from male or female adult C57BL/6J WT, *Ahr*^*-/-*^, or *CypTKO* mice using CD4^+^ Isolation Kit (Miltenyi) in conjunction with QuadroMACS separator (Miltenyi). Media used for cultures was RPMI 1640 (Cell Gro) supplemented with Hepes buffer (Cell Gro), non-essential amino acids (Cell Gro), sodium pyruvate (Cell Gro), penicillin/streptomycin/glutamine (Cell Gro), 2-Mercaptoethanol (Life Technologies) and 5% FBS (Hyclone).

Purified naïve CD4^+^ T cells were plated in 96-well plates at 150,000 cells per well in 100μL and stimulated with plate-coated anti-CD3 (1μg/ml; R&D Systems) at 4°C for 24 hours and by soluble anti-CD28 (1μg/mL, BD) added at time 0. Cells were differentiated under Th17 conditions (human TGF-β (5ng/mL; R&D Systems), murine IL-6 (50ng/mL; R&D Systems)), Th1 conditions (murine IL-12 (10ng/mL; R&D systems), or Treg conditions (human TFG-β 5ng/mL; R&D Systems) for 72 hours (3 days) at 37°C and 5% CO_2_. All cultures included two positive controls, 6-formylindolo[3,2-b] carbazole (FICZ) (200nM; Enzo Life Sciences), which is a tryptophan photoproduct and endogenous high affinity AHR ligand and β-napthoflavone (BNF) another AHR ligand (2μM; Sigma Aldrich). The positive controls were used to determine whether the differentiation cultures were prepared appropriately, and naïve cells responded and differentiated (S1 Fig). All treatments were done in duplicate or triplicate on each 96-well plate.

### OF treatments

Cells were exposed to 8 concentrations of SRM1649b OF (Dr. James Schauer, WSLH, Madison, WI) and solvent control (Dr. James Schauer, WSLH, Madison, WI) added to the culture at time 0. The treatments were in 100μL, making the final volume in each well of the 96-well plate 200μL. The treatment concentrations chosen were based on organic carbon (OC) content because it is extractable and PM, OF, and synthetic PAH mixture treatments can be normalized to it. The highest concentration was 10μg/mL OC and the lowest concentration was 0.00001μg/mL OC. The mass of 15 PAHs, most of which are U.S. EPA priority PAHs, were calculated in nanograms at the highest concentration 10μg/mL OC for the chemically-extracted SRM1649b OF (Table 1, column 2).

**Table 1:** Concentration of 15 PAHs in SRM1649b OF and synthetic SRM1649b PAH mixture and their AHR and CYP activity.

PAHs	ng PAH at 10μg/mL OC SRM1649b OF	ng PAH at 10μg/mL OC synthetic SRM1649b PAH mixture	AHR activators (IEF_B[a]P 6hrs_) [24]	AHR activators (IEF_TCDD 6hrs_) [24]	CYP Inhibitors [14]
Fluoranthene	2.25	2.18	1.05E−2	9.84E−5	X
Benzo[b]fluoranthene	2.23	2.16	8.83	4.90E−2	X
Pyrene	1.80	1.74	7.57E−3	2.59E−5	
Phenanthrene	1.46	1.41	N/A	N/A	
Benzo[ghi]perylene	1.43	1.39	5.47E−3	2.27E−5	
Chrysene	1.10	1.07	3.25	1.41E−2	X
Benzo[e]pyrene	1.07	1.04	2.27E−3	3.71E−5	X
Indeno[1,2,3-cd]pyrene	1.04	1.01	44.20	0.86	
Benzo[a]pyrene	0.89	0.86	1	1.11E−2	X
Benz[a]anthracene	0.76	0.74	0.39	7.64E−7	X
Benzo[k]fluoranthene	0.61	0.60	67.76	0.28	
Perylene	0.22	0.21	N/A	N/A	
Anthracene	0.19	0.18	NI	NI	
Picene	0.14	0.14	0.12	1.90E−3	
Dibenz[a,h]anthracene	0.11	0.10	11.46	0.06	

This table lists 15 PAHs, most of which are U.S. EPA priority PAHs, that are present in SRM1649b PM and its derivatives. The 15 PAHs are ordered starting with the PAH with the highest mass fraction and ending with the PAH with the lowest mass fraction present in SRM1649b. The table shows the mass in nanograms of each of these PAHs at the highest concentration tested, 10μg/mL OC in the OF (column 2) and synthetic PAH mixture (column 3). The table also includes the AHR-inducing potency, represented by induction equivalency factors (IEFs), of the PAHs present in SRM1649b OF and synthetic PAH mixture. The IEFs were derived from effective concentration (EC)_50_ values in a 6-hour luciferase exposure assay using a rat hepatoma cell line [24]. The IEFs are relative to benzo[a]pyrene (B[a]P) a representative carcinogenic PAH (column 4) or 2,3,7,8- tetracholordibenzo-*p*-dioxin (TCDD), the canonical high affinity AHR ligand, at 6 hours (column 5) [24]. For some of the IEFs the information is not available (N/A) or the PAH did not induce AHR activation (NI). Additionally, the CYP inhibition status of the PAHs was included (column 6) [14].

### PAH mixture treatments

Cells were exposed to 8 concentrations of the synthetic SRM1649b PAH mixture (Dr. James Schauer, WSLH, Madison, WI) and Solvent control (Dr. James Schauer, WSLH, Madison, WI) at time 0. The treatments were in 100μL, making the final volume in each well of the 96-well plate 200μL. The treatment concentrations chosen were based on organic carbon (OC) content because it is extractable and PM, OF, and PAH mixture treatments can be normalized to it. The highest concentration was 10μg/mL OC and the lowest concentration was 0.00001μg/mL OC. The mass of 15 PAHs, most of which are U.S. EPA priority PAHs, in nanograms were calculated at the highest concentration 10μg/mL OC for the synthetic SRM1649b PAH mixture (Table 1, column 3).

### Intracellular cytokine staining for T cells

Intracellular cytokine staining for T cells was conducted on day 3, after the T cells were cultured for 72 hours. The cultured cells were stimulated with Cell Stimulation Cocktail (eBioscience) for 5 hours. Brefeldin A 1000X (eBioscience) was added for the final 4.5 hours. Cells were then fixed and permeabilized with Intracellular Fixation & Permeabilization Buffer (eBioscience) or Foxp3/Transcription Factor Staining Buffer Set (eBioscience) and intracellular cytokines overnight at 4°C. T cells were stained with LIVE/DEAD Fixable Blue Dead Cell Stain Kit for UV Excitation (Invitrogen) or Ghost 780 (Tonbo) prior to fixation. Cells were stained with CD4 (BUV395; BD or PE, PeCy5; eBioscience) and TCRB (PeCy7; eBioscience) for extracellular markers. Cells were stained with IL-17A (FITC; eBioscience), KI67 (PE; eBioscience), or FOXP3 (eFluor450; eBioscience). Stained cells were analyzed on Fortessa (BD) or Attune NxT (Invitrogen). Data was analyzed using FlowJo software (TreeStar). Flow plots show cytokine producing cells as percent cytokine producing cells of CD4^+^TCRβ^+^ Live cells or TCRβ^+^ Live cells.

### ELISAs

Supernatant was collected from *in vitro* Th17 cultures and analyzed for mouse IL-17 by ELISA according to manufacturer’s instructions (R&D Systems).

### Quantitative real time PCR (qRT-PCR)

Total RNA was extracted using a RNeasy Mini Kit (Qiagen). cDNA synthesis was completed using SuperScript IV VILO Master Mix (Invitrogen). The qRT-PCR was performed on the Applied Biosystems 7900HT Fast Real-Time PCR system using Taqman Gene Expression Assays and Taqman Universal PCR Mastermix (Life Technologies). Taqman Gene Expression Assays used include: Actin b, Mm02619580_g1; IL-17A, mm00439619_m1; Cyp1A1, Mm00487218_m1. Data were analyzed using the ΔΔCt method with actin serving as the endogenous reference.

### Isolation of bone marrow derived dendritic cells (BMDCs)

Murine bone marrow derived dendritic cells (BMDCs) were isolated from C57BL/6 or *Ahr*^*-/-*^ mice based on a protocol previously described [25]. This isolation protocol isolates primarily BMDCs but includes a small percentage of macrophages. Bone marrow was obtained from the femurs of male or female C57BL/6J mice. Red blood cells (RBCs) were removed using 1mL of RBC lysing solution (eBioscience) for 1–2 minutes and washed twice with complete RPMI media. After washing, cells were counted and 2X10^6^ cells were plated on bacterial culture plates in a total of 10mLs of media. The media was supplemented with 10ng/mL GMCSF and 10ng/mL IL-4. The cells were incubated at 37°C and 5% CO_2_ for 3 days. On day 3 the cells were fed, adding 2.5mLs of complete RPMI media supplemented with GMCSF and IL-4 resulting in a total of 12.5mLs. The cells were cultured for another 2 days at 37°C and 5% CO_2_.

### BMDC culture

On day 5, cells were centrifuged and resuspended to an approximate concentration of 3X10^6^ cells/mL into complete RPMI media. Cells were plated in a 96-well plate with 100uL cells per well for a total of 300,000 cells per well. Cells were treated with 8 concentrations of SRM1649b PM or OF and solvent or phosphate buffered saline (PBS) control as well as 200ng/mL lipopolysaccharide (LPS). Cultures were incubated at 37°C and 5% CO_2_ for 24hours and supernatants and cells were collected.

### OF treatments

Cells were exposed to 8 concentrations of SRM1649b OF (Dr. James Schauer, WSLH, Madison, WI) and Solvent control (Dr. James Schauer, WSLH, Madison, WI) added to the culture at time 0. The treatments were in 100μL, making the final volume in each well of the 96-well plate 200μL. The treatment concentrations chosen were based on organic carbon (OC) content because it is extractable and PM, OF, and PAH mixture treatments can be normalized to it. The highest concentration was 10μg/mL OC and the lowest concentration was 0.00001μg/mL OC.

### PAH mixture treatments

Cells were exposed to 8 concentrations of the synthetic SRM1649b PAH mixture (Dr. James Schauer, WSLH, Madison, WI) and Solvent control (Dr. James Schauer, WSLH, Madison, WI) at time 0. The treatments were in 100μL, making the final volume in each well of the 96-well plate 200μL. The treatment concentrations chosen were based on organic carbon (OC) content because it is extractable and PM, OF, and PAH mixture treatments can be normalized to it. The highest concentration was 10μg/mL OC and the lowest concentration was 0.00001μg/mL OC.

### Intracellular cytokine Staining for BMDCs

Intracellular cytokine staining for BMDCs was conducted on day 6, 24 hours after treatment. The cultured cells were stimulated with Cell Stimulation Cocktail (eBioscience) for 5 hours. Brefeldin A 1000X (eBioscience) was added for the final 4.5 hours. Cells were then fixed and permeabilized with Intracellular Fixation & Permeabilization Buffer (eBioscience) and intracellular cytokines overnight at 4°C. BMDCs were stained with LIVE/DEAD Fixable Blue Dead Cell Stain Kit for UV Excitation (Invitrogen) or Ghost 780 (Tonbo) prior to fixation. Cells were stained with CD11c (PeCy7; eBioscience), F4/80 (PE; eBioscience), CD86 (APC; eBioscience), and MHC II (BV605; eBioscience) for extracellular markers. Cells were stained with IFNγ (eFluor488; eBioscience) and IL-10 (AlexaFluor700). Stained cells were analyzed on Fortessa (BD) or Attune NxT (Invitrogen). Data was analyzed using FlowJo software (TreeStar). Flow plots show cytokine producing cells as percent cytokine producing cells of CD11c^+^F4/80^-^ Live cells.

### Statistics

Statistics were analyzed using GraphPad Prism version 7. For all *in vitro* analyses the question asked was: Is there an interaction between treatment and concentration for the different samples? The two variables tested were treatment and concentration. A test of normality was conducted to determine whether the statistics would be parametric or nonparametric. A 2-way repeated measures analysis of variance (ANOVA) was performed with p value < 0.05. The treatments were SRM1649b, or control and 8 concentrations were tested with the highest concentration being 10μg/mL OC and the lowest concentration 0.00001μg/mL OC.

## Results

### SRM1649b OF enhances Th17 differentiation in an AHR-dependent and CYP- dependent manner *in vitro*

Previously our lab demonstrated that an intact standard ambient urban dust particle, SRM1649b PM, increased IL-17 and CYP1A1 mRNA *in vivo* and enhanced Th17 differentiation in an AHR-dependent manner measured by increased IL-17 mRNA and CYP1A1 mRNA as well as percent IL-17 positive cells in wild-type but not *Ahr* null cells *in vitro* [19]. More recently, we demonstrated that the intact SRM1649b PM enhanced Th17 differentiation in an AHR-dependent manner *in vitro* [26]. The chemically-extracted OF of SRM1649b PM also enhanced Th17 differentiation and increased CYP1A1 mRNA to the same extent as the intact PM suggesting the component of PM driving the responses was present in the OF [19]. In addition to the intact PM and its chemically-extracted OF, individual PAHs, specifically benzo[k]fluoranthene, present in SRM1649b PM and it’s chemically-extracted derivatives enhanced Th17 differentiation in an AHR-dependent manner *in vitro* [19]. PAHs are potent AHR ligands present in SRM1649b at 10^2^ to 10^5^-fold higher than other AHR ligands such as dioxins. Table 1 includes induction equivalency factors (IEFs) based on effective concentration (EC)_50_ values generated from a 6-hour luciferase assay in rat hepatoma cells that measures AHR activation relative to benzo[a]pyrene (B[a]P) (column 4) and to 2,3,7,8-tetracholorodibenzo-*p*-dioxin (TCDD) (column 5) for the 15 PAHs. The IEFs of the PAHs present in PM relative to B[a]P and TCDD demonstrate the differential ability of each PAH to activate the AHR and suggests that some PAHs may be more active in driving AHR-mediated responses than others. Based on this, we hypothesized that PAHs present in SRM1649b enhanced Th17 differentiation via the AHR leading to an effector T cell response. In an attempt to characterize the mechanism in which SRM1649b enhanced Th17 differentiation and the component of PM responsible for the enhanced response, naïve CD4^+^ T cells from WT, C57BL/6J, *Ahr*^-/-^, or CYPTKO mice were exposed to SRM1649b OF or solvent control on day 0 and differentiated under Th17 or Treg conditions for 3 days. Table 1 lists 15 PAHs, most of which are U.S. EPA priority PAHs present in the chemically-extracted OF of SRM1649b starting with the PAH with the highest mass fraction present in SRM1649b down to the lowest. The concentrations presented are the concentrations of each PAH present in the OF at the highest concentration, 10μg/mL OC, tested *in vitro*.

**Fig 1:**
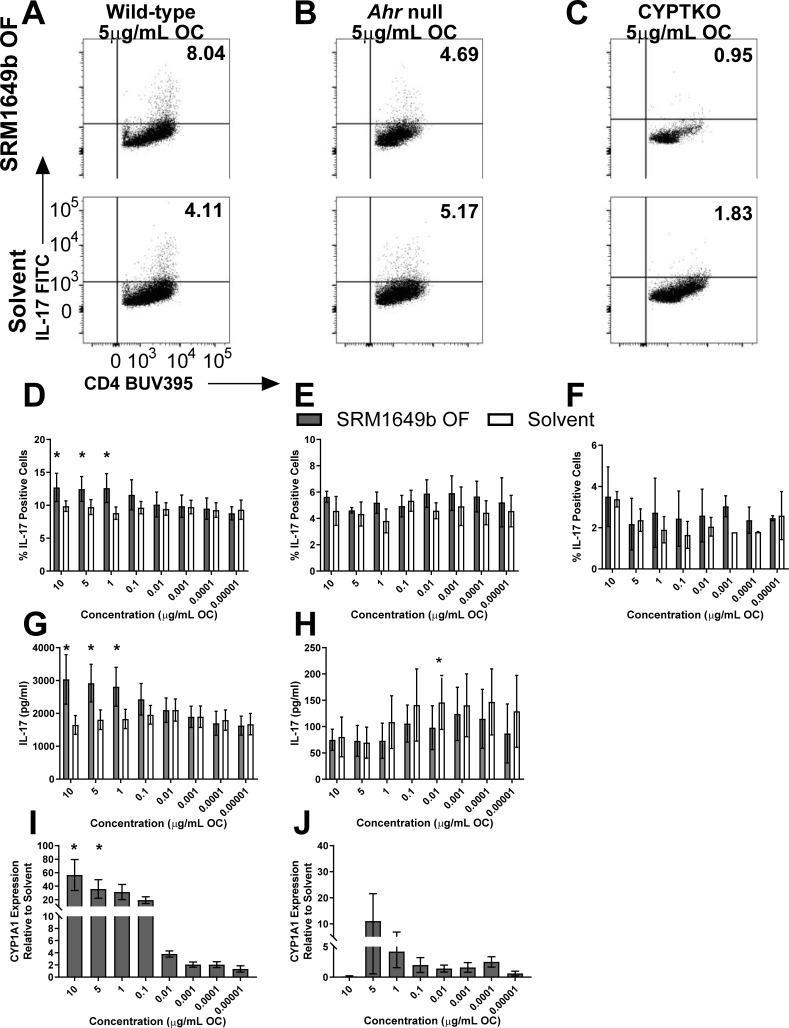
SRM1649b OF enhances Th17 differentiation in an AHR-dependent and CYP- dependent manner *in vitro*. Naïve CD4^+^ T cells were isolated from WT (C57BL/6J), *Ahr*^-/-^, or CYPTKO mice. At time 0, cells were exposed to 8 concentrations of SRM1649b OF or solvent control and cultured for 3 days under Th17 conditions. (A) Representative flow plots of WT Th17 differentiation, measured by percent IL-17 positive cells, at 5μg/mL OC. (B) Representative flow plots of *Ahr*^-/-^ Th17 differentiation at 5μg/mL OC. (C) Representative flow plots of CYPTKO Th17 differentiation at 5μg/mL OC. (D) In WT cells, SRM1649b OF significantly enhanced Th17 differentiation, measured by percent IL-17 positive cells at 10, 5, and 1μg/mL OC compared to solvent control (n = 7). (E) SRM1649b OF had no effect on Th17 differentiation compared to solvent control in *Ahr*^-/-^ cells (n = 3). (F) SRM1649b OF had no effect on Th17 differentiation compared to solvent control in CYPTKO cells (n = 2). (G) In WT cells, SRM1649b OF significantly increased IL-17 production, measured by ELISA, at 10, 5, and 1μg/mL OC (n = 6). (H) In *Ahr*^-/-^ cells, SRM1649b OF suppressed IL-17 production at 0.01μg/mL OC (n = 3). (I) SRM1649b OF increased CYP1A1 mRNA expression at 10 and 5μg/mL OC (n = 4) and (J) had no effect on CYP1A1 mRNA in *Ahr*^-/-^ cells (n = 3). Results are mean ± SEM. Significant differences among groups (*p*<0.05) are indicated by an asterisk.

In C57BL/6J wild-type T cells, SRM1649b OF enhanced Th17 differentiation, measured by increased percent of IL-17 positive cells, at 10, 5, and 1μg/mL OC (Fig 1A and 1D). SRM1649b OF also increased IL-17 protein levels, measured in supernatants at 10, 5, and 1μg/mL OC (Fig 1G). Moreover, to test whether AHR was activated, CYP1A1 mRNA expression was measured and used as a marker for AHR activation. SRM1649b OF significantly increased CYP1A1 mRNA at 10 and 5μg/mL OC (Fig 1I) suggesting that SRM1649b OF activated AHR at high concentrations.

We next tested whether these findings were AHR-dependent. SRM1649b OF did not enhance Th17 differentiation at any of the concentrations tested in *Ahr*^-/-^ cells (Fig 1B and 1E). There was no difference in IL-17 protein levels except at 0.01μg/mL OC in which SRM1649b OF was significantly decreased compared to solvent control in *Ahr*^-/-^ cells (Fig 1H). To ensure there was no AHR activation measured in the *Ahr*^-/-^ T cells, CYP1A1 mRNA was measured and no significant difference was measured between SRM1649b OF and solvent control (Fig 1J).

Cytochrome P450 (CYP) 1A1, CYP1A2, and CYP1B1 enzymes are all canonical downstream targets of AHR activation and are essential for phase 1 metabolism of xenobiotic agents [27, 28]. PAHs are high affinity AHR ligands and are readily metabolized by these CYP enzymes. In addition, some PAHs (Table 1, column 6) have been shown to inhibit CYP enzymes resulting in inhibition of their own metabolism as well as other organic constituents present in the mixture [14, 15, 29]. To test the hypothesis that PAHs present in SRM1649b OF required metabolism to enhance Th17 differentiation, naïve CD4^+^ T cells were isolated from CYPTKO mice and differentiated under Th17 conditions. Overall there was a reduction in the percent of IL-17 positive cells generated in CYPTKO cells regardless of treatment, suggesting that cells lacking CYP enzymes have a metabolism deficiency and do not differentiate as well as wild-type. There was no effect of SRM1649b OF on Th17 differentiation in CYPTKO cells (Fig 1C and 1F) suggesting that metabolism is required to elicit enhanced Th17 differentiation by SRM1649b OF.

### SRM1649b suppresses Treg differentiation at low concentrations *in vitro*

**Fig 2:**
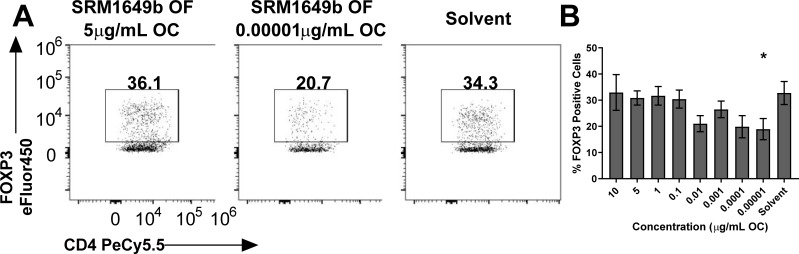
SRM1649b suppresses Treg differentiation at low concentrations *in vitro*. Naïve CD4^+^ T cells were isolated from WT (C57BL/6J), *Ahr*^-/-^, or CYPTKO mice. At time 0, cells were exposed to 8 concentrations of SRM1649b OF or solvent control and cultured for 3 days under Treg conditions. (A) Representative flow plots of WT Treg differentiation, measured by percent FOXP3 positive cells, at 5μg/mL OC (left) and 0.00001μg/mL OC (right), (B) SRM1649b OF significantly suppressed Treg differentiation at 0.00001μg/mL OC (n = 4). Results are mean ± SEM. Significant differences among groups (*p*<0.05) are indicated by an asterisk.

The AHR plays a critical role in maintaining the balance between effector T cell responses and regulatory T cell responses [16–18, 30]. *Ahr* is most highly expressed in Th17 cells and is moderately expressed in T regulatory type 1 (Tr1) cells and forkhead box P3 (FOXP3) ^+^ Treg cells. Given the role of AHR in modulating Th17 and Treg differentiation we tested the ability of SRM1649b OF to enhance FOXP3^+^ Treg differentiation. SRM1649b OF had no effect on Treg differentiation at high concentrations but suppressed Treg differentiation at 0.00001μg/mL OC compared to solvent control (Fig 2A and 2B).

### SRM1649b OF increases IFNγ positive BMDCs in an AHR-dependent manner and is required for maintenance of IL-10 positive BMDCs *in vitro*

**Fig 3:**
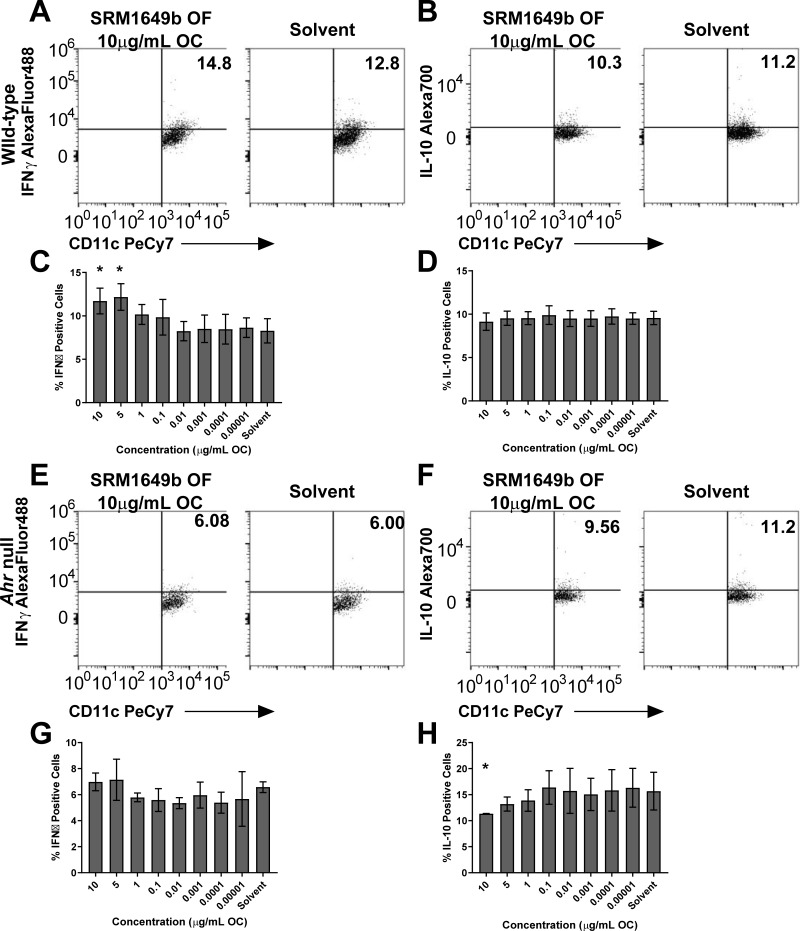
SRM1649b OF increases IFNγ positive BMDCs in an AHR-dependent manner and generates IL-10 positive BMDCS in an AHR-dependent manner *in vitro*. BMDCs were isolated from femurs of WT (C57BL/6J) or *Ahr*^-/-^ mice. The cells were cultured on bacterial culture plates in complete RPMI media supplemented with GMCSF (10ng/mL) and IL-4 (GMCSF) for 3 days at 37°C and 5% CO_2_. On day 3, an additional 2.5mLs of media supplemented with GMCSF and IL-4 was added to the culture for an additional 2 days. On day 5, the cells were harvested, resuspended in complete RPMI media, and plated in 96-well plates in the presence of 200ng/mL LPS plus 8 concentrations of SRM1649b OF or solvent control. (A) Representative flow plots of WT IFNγ positive BMDCS, measured by F4/80^-^CD11c^+^IFNγ^+^ cells. (B) Representative flow plots of WT IL-10 positive BMDCS, measured by F4/80^-^CD11c^+^IL-10^+^ cells. (C) SRM1649b OF significantly increased the percent of WT IFNγ positive BMDCs at 10 and 5μg/mL OC (n = 6). (D) There was no significant difference in the percent of IL-10 positive BMDCS between SRM1649b OF and solvent control in WT cells (n = 6) (E) Representative flow plots of *Ahr*^-/-^ IFNγ positive BMDCS. (F) Representative flow plots of *Ahr*^-/-^ IL-10 positive BMDCS. (G) In *Ahr*^-/-^ cells, there was no significant difference in percent of IFNγ positive BMDCS (n = 2) and (H) significant suppression of IL-10 positive BMDCs at 10μg/mL OC (n = 2). Results are mean ± SEM. Significant differences among groups (*p*<0.05) are indicated by an asterisk.

DCs are the key APCs that contribute to T cell activation and differentiation. In addition to playing a role in T cells, AHR has also been shown to play an important role in dendritic cells [31]. Given this, we tested whether SRM1649b OF influenced the cytokine production of bone marrow derived dendritic cells (BMDCs) *in vitro*. BMDCs were isolated from femurs of C57BL/6J wild-type mice and *Ahr*^-/-^ and cultured in the presence of IL-4 and GMCSF to promote growth of DCs rather than macrophages. After 5 days of culture, the cells were harvested, counted, plated, and treated with SRM1649b OF or solvent control as well as 200ng/mL of LPS for 24 hours. After 24 hours, BMDCs were harvested for flow cytometry. The BMDCs were selected as the CD11c^+^F4/80^-^ population. To determine whether inflammatory or tolerogenic DCs were generated we measured IFNγ as an inflammatory marker and IL-10 as a marker for regulation. SRM1649b OF significantly increased the percent of IFNγ positive cells at 10 and 5μg/mL compared to solvent control (Fig 3A and 3C) and had no effect on the percent of IL-10 positive cells (Fig 3B and 3D).

Next, we tested whether the increased percent in IFNγ positive BMDCs was AHR-dependent using BMDCs isolated for *Ahr*^-/-^ null mice. SRM1649b OF did not increase the percent of IFNγ positive cells compared to solvent control (Fig 3E and 3G). Additionally, there was a significant suppression of the percent of IL-10 positive BMDCs treated with SRM1649b OF compared to solvent control at 10μg/mL OC suggesting AHR is important in the maintenance of IL-10 positive BMDCs (Fig 3F and 3H).

### Synthetic SRM1649b PAH mixture enhances Th17 differentiation in an AHR-dependent manner and CYP-independent manner *in vitro*

**Fig 4:**
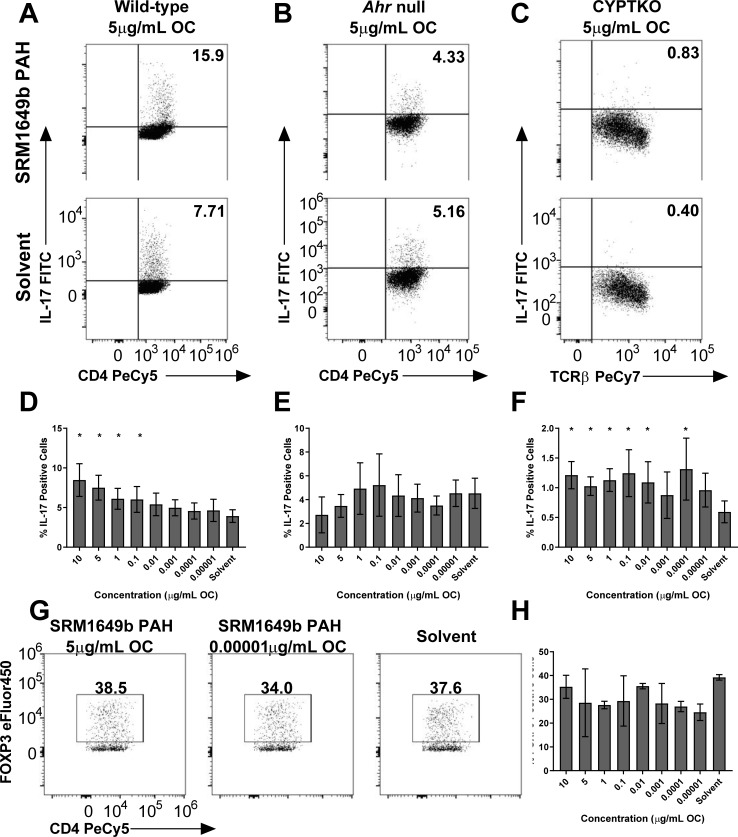
SRM1649b PAH mixture enhances Th17 differentiation in an AHR-dependent and CYP- independent manner *in vitro* and has no effect on Treg differentiation. Naïve CD4^+^ T cells were isolated from WT (C57BL/6J), *Ahr*^-/-^, or CYPTKO mice. At time 0, cells were exposed to 8 concentrations of SRM1649b PAH mixture or solvent control and cultured for 3 days under Th17 conditions. (A) Representative flow plots of WT Th17 differentiation, measured by percent IL-17 positive cells, at 5μg/mL OC. (B) Representative flow plots of *Ahr*^-/-^ Th17 differentiation at 5μg/mL OC. (C) Representative flow plots of CYPTKO Th17 differentiation at 5μg/mL OC. (D) In WT cells, SRM1649b PAH mixture significantly enhanced Th17 differentiation, measured by percent IL-17 positive cells at 10, 5, 1 and 0.1μg/mL OC compared to solvent control (n = 7). (E) SRM1649b PAH mixture had no effect on Th17 differentiation compared to solvent control in *Ahr*^-/-^ cells (n = 4). (F) In CYPTKO cells, SRM1649b PAH mixture significantly increased Th17 differentiation compared to solvent control at all concentrations tested except 0.001 and 0.00001μg/mL OC (n = 3). (G) Representative flow plots of WT Treg differentiation at 5μg/mL OC (left) and 0.00001μg/mL OC (middle). (H) There was no significant difference in the percent of FOXP3 positive cells between SRM1649b PAH mixture and solvent control (n = 2). Results are mean ± SEM. Significant differences among groups (*p*<0.05) are indicated by an asterisk.

We hypothesized that PAHs present in SRM1649b enhanced Th17 differentiation via the AHR leading to an effector T cell response. To test this, PAH mixtures that replicate the PAH milieu of 15 PAHs, most of which are U.S. EPA priority PAHs present in the OF extracts of SRM1649b were synthesized and assayed in the naïve CD4 T cell assay under Th17 differentiation conditions. Table 1 lists 15 PAHs, most of which are U.S. EPA priority PAHs, present in the synthetic PAH mixture of SRM1649b starting with the PAH with the highest mass fraction present in SRM1649b down to the lowest. The concentrations presented are the concentrations of each PAH present in the synthetic PAH mixture at the highest concentration, 10μg/mL OC, tested *in vitro*. Synthetic SRM1649b PAHs enhanced Th17 differentiation at 10, 5, 1, and 0.1μg/mL OC (Fig 4A and 4D).

In *Ahr*^-/-^ naïve T cells, the synthetic SRM1649b PAH mixture had no effect on Th17 differentiation compared to solvent control (Fig 4B and 4E) suggesting that enhanced Th17 differentiation by SRM1649b PAH mixture was dependent on the AHR. In CYPTKO naïve T cells, the synthetic SRM1649b PAH mixture enhanced Th17 differentiation at 10, 5, 1, 0.1, 0.01, and 0.0001μg/mL OC (Fig 4C and 4F) suggesting that metabolism of PAHs is not required to elicit the effect of SRM1649b PAH mixtures.

### Synthetic SRM1649b PAH mixture has no effect on Treg differentiation *in vitro*

Given that AHR is important in the balance of Th17 and Treg cells, and SRM1649b OF suppressed Treg differentiation at low concentrations, we tested whether the synthetic SRM1649b PAH mixture suppressed Treg differentiation. The synthetic SRM1649b PAH mixture was assayed in the naïve CD4 T cell assay under Treg differentiation conditions. The synthetic SRM1649b PAH mixture had no effect on Treg differentiation at high or low concentrations (Fig 4G and 4H).

### Synthetic SRM1649b PAH mixture increases percent of IFNγ positive BMDCs *in vitro*

**Fig 5:**
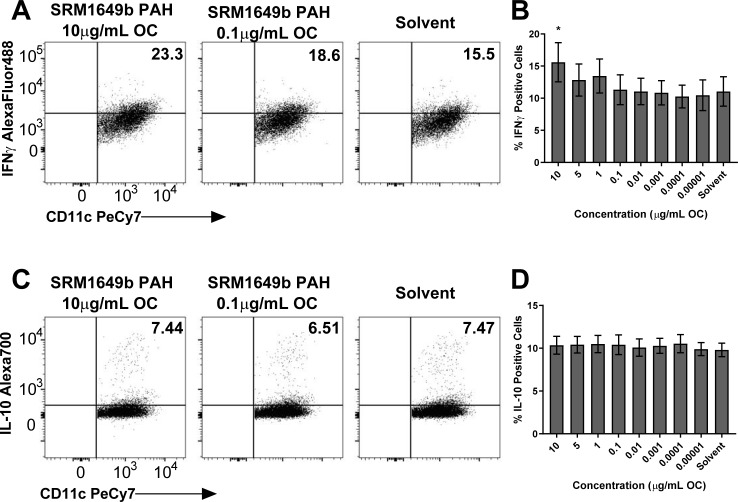
SRM1649b PAH mixtures increase percent of IFNγ positive BMDCs *in vitro*. BMDCs were isolated from femurs of WT (C57BL/6J). The cells were cultured on bacterial culture plates in complete RPMI media supplemented with GMCSF (10ng/mL) and IL-4 (GMCSF) for 3 days at 37°C and 5% CO_2_. On day 3, an additional 2.5mLs of media supplemented with GMCSF and IL-4 was added to the culture for an additional 2 days. On day 5, the cells were harvested, resuspended in complete RPMI media, and plated in 96-well plates in the presence of 200ng/mL LPS plus 8 concentrations of SRM1649b PAH mixture or solvent control. (A) Representative flow plots of IFNγ positive BMDCS, measured by F4/80^-^CD11c^+^IFNγ^+^ cells at 10μg/mL OC. (B) SRM1649b PAH mixture significantly increased the percent of IFNγ positive BMDCs at 10μg/mL OC (n = 6). (C) Representative flow plots of IL-10 positive BMDCS, measured by F4/80^-^CD11c^+^IFNγ^+^ cells at 10μg/mL OC. (D) There was no significant difference in the percent of IL-10 positive BMDCS between SRM1649b PAH mixture and solvent control in WT cells (n = 6). Results are mean ± SEM. Significant differences among groups (*p*<0.05) are indicated by an asterisk.

We next tested the effect of the synthetic SRM1649b PAH mixture on BMDCs. The synthetic SRM1649b PAH mixture increased the percent of IFNγ positive cells (Fig 5A and 5B) and had no effect on percent of IL-10 positive cells (Fig 5C and 5D), similar to SRM1649b OF. This suggests that the PAHs may be responsible for the increase in IFNγ production by BMDCs.

## Discussion

The current study investigated the effects of PAHs present in PM on T cells and BMDCs *in vitro*. In addition, this study aimed to identify the pathway(s) responsible for driving the proinflammatory responses. We hypothesized that PAHs present in PM activate the AHR and drive proinflammatory responses in T cells and BMDCs. Our results support that PAHs present in PM contribute to immune responses by acting directly on T cells and enhancing Th17 differentiation through the AHR/CYP pathway and directly on DCs increasing the percent of IFNγ positive DCs through the AHR pathway. Similarly, another group recently published that PM enhances DC activation and enhances naïve T cell differentiation towards a Th17-like phenotype in an AHR-dependent manner *in vitro* and *in vivo* [32]. In addition, previous publications have demonstrated that PAHs present in the neutral fraction of PM and it’s chemically-extracted OF are the primary contributors to the AHR-mediated activity [33] and induction of AHR target genes was not antagonized by other components of complex mixtures, since induction of CYP1A1, CYP1B1 and TIPARP transcripts reached maximum levels induced by PAHs [34].

Our results showed that SRM1649b OF enhanced Th17 differentiation in an AHR-dependent and CYP-dependent manner. In addition, SRM1649b OF increased IFNγ positive BMDCs in an AHR-dependent manner. SRM1649b OF treated BMDCs required AHR for the generation of IL-10 positive BMDCs. Additionally, SRM1649b OF suppressed Treg differentiation at low concentrations. These data demonstrate that PAHs present in PM enhance pro-inflammatory responses, and this is driven by AHR activation, but also by CYP metabolizing enzymes. CYPs convert PAHs to more polar and water-soluble metabolites for excretion, however during the metabolism, unstable reactive intermediates of PAHs are formed, and these metabolites cause cell toxicity, DNA damage, and lead to the carcinogenicity [13, 15]. Recently, it was shown that although PAHs are major contributors to the AHR-mediated activity of organic compounds associated with diesel exhaust particles, polar compounds, which include PAH metabolites, present in these mixtures are more active in human cells, as compared with rodent cells [35]. In addition, they demonstrated that benzo[k]fluoranthene was the most potent AHR agonist in human cells [35]. Based on this, CYP enzymes may be metabolizing PAHs present in SRM1649b OF and reactive intermediates may be leading to enhanced Th17 differentiation and thus the enzymes themselves are required to metabolize the PAHs to drive the enhanced effect. Recently, it was shown that the intact SRM1649b PM enhanced Th17 differentiation in an AHR-dependent manner, but using a murine model of autoimmune disease, was shown to reduce pathologic T cells in the CNS *in vivo* [26]. These results could be explained by differences in metabolism and bioavailability of the PAHs when adhered to particles as opposed to the OF which could make PAHs more bioavailable. Future studies should focus on understanding the role of AHR and CYP metabolism in driving proinflammatory T cell and DC effects mediated by the chemically-extracted OF and synthetic PAH mixtures of SRM1649b.

The current study also addressed whether individual components of PM, specifically PAHs, drive proinflammatory T cells and BMDCs through the AHR/CYP pathway. A synthetic mixture of PAHs was created based on 15 of the PAHs present in SRM1649b OF with the concentrations representing the concentration present at the highest concentration used *in vitro*, 10μg/mL OC. The synthetic SRM1649b PAH mixture had no effect on Treg differentiation (Fig 5G and 5H), but enhanced Th17 differentiation in an AHR-dependent, CYP-independent manner (Fig 5A and 5D WT, 5B and 5E AHR^-/-^ and 5C and 5F CYP). Some PAHs are potent CYP inhibitors (Table 1 column 6) and therefore individual PAHs can affect their own metabolism as well as the metabolism of other PAHs [14, 29]. The PAHs present in SRM1649b are present at different concentrations and some are more potent activators of the AHR, such as benzo[b]fluoranthene and benzo[k]fluoranthene (Table 1 column 4 and 5), which may suggest that some PAHs contribute more to the observed response than others. SRM1649b OF required CYP metabolism to elicit inflammatory effects, but SRM1649b PAH mixtures do not. One explanation is that the PAHs in the OF, with other organic constituents, elicit synergistic or antagonistic effects and do not inhibit CYPs and thus metabolism occurs. With the PAH mixture, the PAHs are the only organic constituents present to elicit their effects and ultimately inhibit the CYP enzymes. When the CYPs are genetically absent, as in the CYPTKO, the synthetic SRM1649b PAH mixture still enhanced Th17 differentiation because the CYPs are not required for the response. Another possible explanation is that synergistic and antagonistic effects of other organic constituents present in SRM1649b OF prevent the PAHs from activating the AHR with the same potency and metabolism of these organic constituents, not PAHs, is required to allow the PAHs to activate AHR and enhance Th17 differentiation. In CYPTKO cells, all metabolism is blocked, and the synergistic and antagonistic effects of the organic constituents remains intact preventing the PAHs from strongly activating AHR and enhancing Th17 differentiation. In the synthetic SRM1649b PAH mixture, all other organic constituents that have the potential to elicit synergistic and antagonistic effects on the PAHs are absent and therefore CYP enzymes are not required because the PAHs do not require metabolism to enhance Th17 differentiation.

## Conclusions

Overall, these results demonstrate that PAHs present in PM, and its chemically-extracted OF, are active components of PM that have the capacity to shift the immune balance between regulation and inflammation. Moreover, AHR activation by PAHs and CYP metabolism of PAHs alter immune responses and specific nature of the mixtures can alter the pathway responses. These results exemplify the complicated nature of PM, with its multiple components synergistically and/or antagonistically effecting immune responses, and the need for further studies identifying how individual components, PAHs or other active components, and mixtures of those components shift the balance between regulatory and inflammatory responses through the AHR and CYP metabolism. In addition, further studies need to understand how the chemically-extracted OF and synthetic PAH mixtures of SRM1649b shift the balance between regulatory and inflammatory responses through AHR and CYP metabolism using *in vivo* models. Identification of specific PAHs or specific pathway components and more importantly investigating these effects *in vivo* in physiologically relevant systems could allow for targeted therapies to shift the balance from proinflammatory to regulatory and mitigate disease.

## Supporting information

S1 Fig Positive controls for T cell differentiation. The positive controls used for T cell differentiation are AHR ligands, FICZ and BNF, that are known to enhance T cell responses. This figure shows the percent of IL-17 positive cells measured by flow cytometry for solvent control, FICZ treatment, and BNF treatment. Addition of FICZ increases the percent of IL-17 positive cells as does the addition of BNF. The positive controls are used to tell if the naïve CD4 T cells differentiated appropriately. If no increase is seen with FICZ or BNF, the experiment is not included in analysis. Abbreviations: FICZ, 6-formylindolo[3,2-b] carbazole; BNF, β-naphthoflavone.
(EPS)

S1 Table List of PAHs present in SRM1649b. This table lists the PAHs present in SRM1649b and are ordered starting with the PAH with the highest mass fraction and ending with the PAH with the lowest mass fraction (top to bottom).
(DOCX)
