# Supplementary material for: Polycyclic aromatic hydrocarbons (PAHs) present in ambient urban dust drive proinflammatory T cell and dendritic cell responses via the aryl hydrocarbon receptor (AHR) in vitro
Source: PLoS One. 2018 Dec 21;13(12):e0209690. doi: 10.1371/journal.pone.0209690 (PMC6303068; doi:10.1371/journal.pone.0209690)
Supplement: S1 Table — This table lists the PAHs present in SRM1649b and are ordered starting with the PAH with the highest mass fraction and ending with the PAH with the lowest mass fraction (top to bottom). (DOCX) [file pone.0209690.s002.docx]

| **PAHs in SRM1649b** | |
| --- | --- |
| Fluoranthene | 1-Methylpyrene |
| Benzo[b]fluoranthene | Dibenzo[j,l]fluoranthene |
| Pyrene | 2-Methylchrysene |
| Phenanthrene | Cyclopenta[cd]pyrene |
| Benzo[ghi]perylene | Benzo[b]chyrsene |
| Coronene | 1-Methylphenanthrene |
| Chrysene | 3-Methylfluoranthene |
| Benzo[e]pyrene | Dibenz[a,j]anthracene |
| Indeno[1,2,3-cd]pyrene | Dibenz[a,h]anthracene |
| Benzo[a]pyrene | 4H-Cyclopenta[def]phenanthrene |
| Benz[a]anthracene | Dibenzo[e,l]pyrene |
| Benzo[j]fluoranthene | 4-Methylpyrene |
| Benzo[k]fluoranthene | Retene |
| Triphenylene | 8-Methylfluoranthene |
| 2-Methylnaphthalene | 3-Methylchrysene |
| Naphthalene | Fluorene |
| Benzo[ghi]fluoranthene | 1,7-Dimethylphenanthrene |
| 2-Methylphenanthrene | Dibenz[a,c]anthracene |
| Dibenzo[b,k]fluoranthene | Pentaphene |
| Naphtho[1,2-b]fluoranthene | Acenaphthene |
| Perylene | Dibenzothiophene |
| Dibenzo[a,e]pyrene | Acenaphthylene |
| 3-Methylphenanthrene | Biphenyl |
| 2-Methylpyrene | Naphtho[2,3-e]pyrene( |
| Anthanthrene | Naphtho[2,3-b]fluoranthene |
| Benzo[c]phenanthrene | 1-Methylfluoranthene |
| Anthracene | Benzo[c]chyrsene |
| Picene | Dibenzo[b,e]fluoranthene |
| Benzo[a]fluoranthene | Dibenzo[a,k]fluoranthene |
| Naphtho[2,1-a]pyrene | Dibenzo[a,l]pyrene |
